# Representativeness of the LifeLines Cohort Study

**DOI:** 10.1371/journal.pone.0137203

**Published:** 2015-09-02

**Authors:** Bart Klijs, Salome Scholtens, Jornt J. Mandemakers, Harold Snieder, Ronald P. Stolk, Nynke Smidt

**Affiliations:** 1 Department of Epidemiology, University of Groningen, University Medical Center Groningen, Groningen, the Netherlands; 2 LifeLines Cohort Study and Biobank, Groningen, the Netherlands; 3 Sociology of Consumption and Households, Wageningen University, Wageningen, the Netherlands; University of Oxford, UNITED KINGDOM

## Abstract

**Background:**

LifeLines is a large prospective population-based three generation cohort study in the north of the Netherlands. Different recruitment strategies were adopted: recruitment of an index population via general practitioners, subsequent inclusion of their family members, and online self-registration. Our aim was to investigate the representativeness of the adult study population at baseline and to evaluate differences in the study population according to recruitment strategy.

**Methods:**

Demographic characteristics of the LifeLines study population, recruited between 2006–2013, were compared with the total adult population in the north of the Netherlands as registered in the Dutch population register. Socioeconomic characteristics, lifestyle, chronic diseases, and general health were further compared with participants of the Permanent Survey of Living Conditions within the region (2005–2011, N = 6,093). Differences according to recruitment strategy were assessed.

**Results:**

Compared with the population of the north of the Netherlands, LifeLines participants were more often female, middle aged, married, living in a semi-urban place and Dutch native. Adjusted for differences in demographic composition, in LifeLines a smaller proportion had a low educational attainment (5% versus 14%) or had ever smoked (54% versus 66%). Differences in the prevalence of various chronic diseases and low general health scores were mostly smaller than 3%. The age profiles of the three recruitment groups differed due to age related inclusion criteria of the recruitment groups. Other differences according to recruitment strategy were small.

**Conclusions:**

Our results suggest that, adjusted for differences in demographic composition, the LifeLines adult study population is broadly representative for the adult population of the north of the Netherlands. The recruitment strategy had a minor effect on the level of representativeness. These findings indicate that the risk of selection bias is low and that risk estimates in LifeLines can be generalized to the general population.

## Introduction

LifeLines is an observational cohort study aiming to unravel the interaction between genetic and environmental factors in the development of multifactorial diseases [1]. The recruitment of more than 165 000 persons from the three northern provinces of the Netherlands, which is 10% of the population, was performed between 2006 and 2013 [2]. During this period, participants completed a number of questionnaires, underwent a physical examination, and biological samples including DNA were collected [1,2]. Participants will be followed up with standardized measurements for at least thirty years [1,2].

To include three generations of participants, a three generation design and recruitment strategy was adopted [1,2]. Firstly, an index population aged 25–49 years was recruited via participating general practitioners (GPs). Subsequently, older and younger family members were invited by LifeLines to take part. In addition, adults could self-register to participate via the LifeLines website. The three generation design has several advantages [1]. Among others, it allows separating between genetic and non-genetic factors in familial transmission of diseases and it provides opportunities to study health effects of family factors and socio-economic mobility [1]. In addition, we expect that family ties within the cohort will help to limit non-response and attrition [1].

A major concern in population based cohort studies is selection bias. Selection bias is the systematic error induced if the association between exposure and disease differs for those who participate and those who do not participate [3]. This means that the risk for selection bias may be particularly high when a subgroup of the population with a specific risk profile is strongly unrepresented. Previous studies show that individuals who are old, single, immigrant, or have a low socioeconomic status are less inclined to participate in large population based cohort studies [4–7]. Furthermore, participants to cohort studies often have less chronic diseases and have a better level of functioning than those who do not participate [4–6].

The primary aim of this paper is to investigate to what extent the LifeLines study population aged 18 and older is representative for the adult population in the northern provinces of the Netherlands. Representativeness on demographic characteristics is assessed by comparing LifeLines with the complete adult population of the northern provinces of the Netherlands using data from the Dutch population register. Representativeness on socioeconomic characteristics, lifestyle factors, the prevalence of chronic diseases and general health is further assessed by comparing with the Permanent Survey of Living Conditions (POLS). This annual cross-sectional survey is carried out by Statistics Netherlands and is a main source of representative information on various aspects of the living situation in the Netherlands [8]. In addition, we investigate differences in the LifeLines study population according to recruitment strategy.

## Materials and Methods

### LifeLines study population

In the LifeLines Cohort Study, a recruitment strategy was adopted that aimed to include three generations of participants. Firstly, all GPs in the three northern provinces of the Netherlands were invited to participate and asked to invite their registered patients aged 25–49 years. Patients who were unable to read Dutch or who had limited life expectancy due to severe illness were excluded by the GP and not invited for participation. Participants who gave written informed consent were included as the “index population”. Subsequently, all persons in the index population were asked to indicate whether family members (partner, parents, parents-in-law, and children) could be invited and to provide their contact details. Family members were invited by LifeLines; those who gave their informed consent were included in the study as “family member”. Furthermore, persons aged 18 years and older could participate in this study through “self-registration” via the LifeLines website. These self-registrants were also asked to invite family members as outlined above. LifeLines aimed to include three generations of participants, but individuals who had no family member participating in the study were not excluded. Although the inclusion started in 2006 and ended in 2013, most participants (57%) were included in the last two years.

All participants aged 18 years and older were asked to complete a comprehensive questionnaire covering the occurrence of diseases, general health, lifestyle, diet, physical activity, personality, social support, medication use and more. In addition, all participants aged 18 years and older were invited to one of the 10 research sites within the region where a number of measurements were performed covering anthropometry, blood pressure, pulmonary function, heart function (electrocardiogram) and cognition [2,9]. In addition, a fasting blood sample was taken, 24 hour urine was collected, and psychiatric disorders were assessed in an interview with one of the research nurses [2,10].

All participants signed an informed consent form before they received an invitation for the physical examination. The LifeLines Cohort Study is conducted according to the principles of the Declaration of Helsinki and in accordance with research code University Medical Center Groningen (UMCG). The LifeLines study is approved by the medical ethical committee of the UMCG, the Netherlands. For a comprehensive overview of the data collection, please visit the LifeLines catalogue at www.LifeLines.net.

### Source population

To investigate to what extent the individuals in LifeLines aged 18 and older are representative, we compared specific characteristics of the LifeLines participants with the complete adult population in the three northern provinces of the Netherlands at May 1^st^ 2012. We opted for this reference date, as half of the total study population had been included at this point in time. Representativeness of children in LifeLines was not evaluated since this requires a different approach and a different set of indicators. Information on age, sex, marital status, urbanization level, and the country of origin of both of the parents was available for the entire population in the northern provinces of the Netherlands through the Dutch population register. The Dutch population register is the backbone of the Dutch governments’ population administration and contains administrative information of all inhabitants of the Netherlands. It has been shown that 98% of the addresses and persons in the register are recorded correctly [11]. The data used for our analysis is available through Statistics Netherlands.

Information on the highest completed level of education, participation in paid work, body weight and height, smoking behavior, the occurrence of specific chronic diseases and general health according to the 12 item short form health survey (SF-12) was available for a representative sample through the Dutch POLS-survey [7,12]. This national annually repeated cross-sectional survey is carried out by Statistics Netherlands and is the main source of representative information on various aspects of the living situation in the Dutch non-institutionalized population [8]. The subjects approached for the survey are identified on the basis of probability sampling from the population register of the Netherlands. Non-response in the survey is 35–40%. Information on education, work, self-reported weight, self-reported height, and smoking behavior was collected in a face-to-face interview and information on chronic diseases and general health in a written questionnaire. Individuals who lived in the northern part of the Netherlands at May 1^st^ 2012 and participated in the survey between 2005–2011 were selected using a personal identification number contained in the Dutch population register and POLS. When persons participated more than once, the most recent year was selected. Persons in POLS with missing information on any of the variables were excluded from the analysis.

### Demographics and socioeconomic position

The definition of western and non-western immigrants was based on the father’s and mother’s country of birth according to the definition of Statistics Netherlands. The level of urbanization was defined on the basis of the area address density. An area address density of <1000 addresses/km^2^ was considered rural, 1000–1500 addresses/km^2^ was semi urban, and > = 1500 addresses/km^2^ was urban. The highest completed level of education was categorized as elementary, secondary or tertiary. A dichotomous variable (yes/no) was created indicating whether or not persons were involved in paid work (i.e. performed one or more hours of paid work) [13].

### Lifestyle, disease and general health indicators

Body weight and height were used to calculate BMI, which was categorized as underweight (<18.5 kg/m^2^), normal weight (18.5–24.9 kg/m^2^), overweight (25–29.9 kg/m^2^) and obese (> = 30 kg/m^2^). Smoking behavior was labeled as ever or never. Dichotomous variables were created for the occurrence of diabetes mellitus, stroke, myocardial infarction, cancer, chronic non-specific lung disease, osteoarthritis, rheumatoid arthritis and psoriasis. Furthermore, an indicator of self-assessed health that was less than good was created [12]. Dichotomous variables were created indicating moderate or severe limitations on four domains of physical functioning and on two domains of emotional functioning of the SF-12 [12]. Lastly, a variable was constructed representing rather much or very much interference of pain with normal work [10].

### Data analysis

To assess to what extent the LifeLines cohort is representative with respect to age and sex, a population pyramid was plotted for the LifeLines population in comparison with the population of the northern provinces of the Netherlands. For all other variables, crude and standardized percentages were compared with percentages in the Dutch population register and POLS. Direct standardization to the adult population in the northern provinces of the Netherlands at May 1^st^ 2012 was used. We standardized for age (18–29, 30–39, 40–49, 50–59, 60–69, 70–79, > = 80 years), sex, marital status (married or registered partner; yes/no), and urbanization (Rural/(Semi-)Urban). Percentages for marital status and urbanization level were standardized for age and sex only. We decided not to standardize for migrant status because low percentages of migrants in the oldest age groups produced empty cells in the standardization procedure. Individuals in LifeLines who had information missing on the items used for standardization were excluded from the analysis (also see results). Missing values on other items were omitted per item. To assess to what extent the index population, family members and self-registrants differed, we compared crude and standardized percentages in these three subsamples.

## Results

### Data samples

LifeLines consists of 167 729 participants, of which 152 915 (91.2%) were adults. Of the adult participants 1434 (0.9%) had information missing on marital status (N = 1192, 0.8%) or urbanization level (N = 242, 0.2%) and were excluded from further analysis. This yielded a study population of 151 481 individuals covering 81 840 (54.0%) individuals recruited by GPs (index population), 48 366 (31.9%) family members and 21 275 (14.0%) self-registrants. Non-response was lower than 3%, except for ‘no paid job’ (4.7%) and ‘ever smoking’ (7.1%). According to the Dutch population register, May 1^st^ 2012, 1 235 537 persons aged 18 or older lived in the three northern provinces of the Netherlands. Of these individuals 6093 participated in the POLS survey between 2005–2011 and completed the oral interview, while 4494 persons completed the written questionnaire.

### LifeLines versus Dutch population register

**Fig 1:**
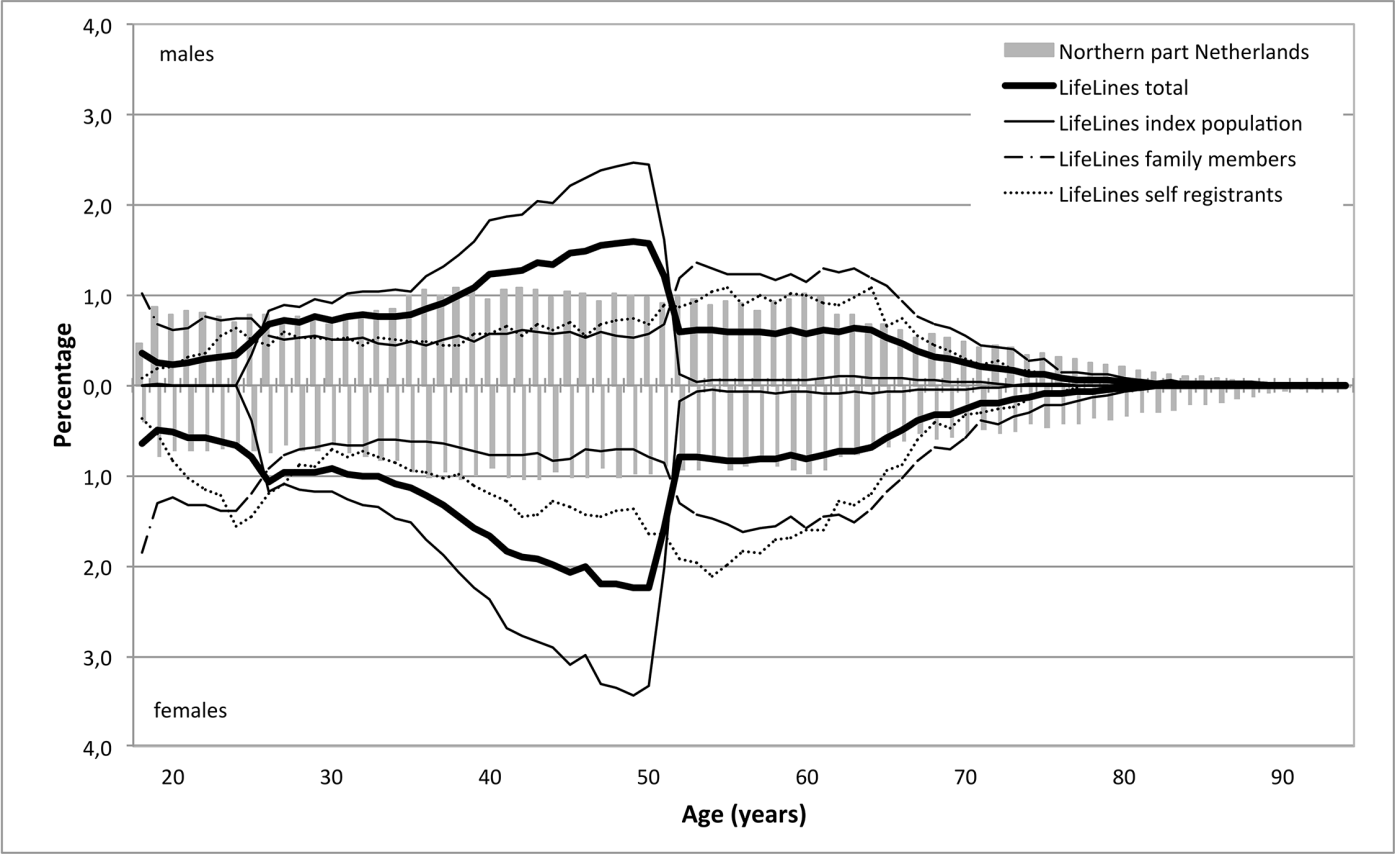
Age distribution within LifeLines as compared with the population of the north of the Netherlands.

In LifeLines there was an overrepresentation of men aged 39–51 and women aged 24–51 (Fig 1). Women were overrepresented (58.5% in LifeLines versus 50.7% in Dutch population register; Table 1). Comparing the standardized percentages showed an overrepresentation of individuals who are married or have a registered partner (60.0% versus 57.5%) or live in a semi-urban place (21.9% versus 18.1%). There was an underrepresentation of western- (4.1% versus 6.4%) and non-western immigrants (1.3% versus 3.2%).

**Table 1:** Demographic characteristics of the LifeLines study population as compared with the population of the three northern provinces of the Netherlands.

	LifeLines		Permanent Survey of Living Conditions (sample)		Dutch population register, (total population north of the Netherlands)
	Crude %	Standardized %	Crude %	Standardized %	%
N	151,481		6,093		1,235,537
**Demographic indicators**					
Women	58.5		51.3		50.7
18–24 years	5.9		9.1		9.9
25–49 years	62.4		45.5		45.5
50–74 years	30.3		39.0		37.4
> = 75 years	1.4		6.5		7.2
Married or registered partner	62.2	60.0	60.6	57.5	57.5
Urban municipality	13.9	15.1	15.3	16.4	16.0
Semi urban municipality	21.9	21.9	18.5	18.6	18.1
Rural municipality	64.1	63.1	66.2	65.0	65.9
Western migrant	4.1	4.1	5.9	5.9	6.4
Non-Western migrant	1.5	1.3	1.8	2.0	3.2

Direct standardization for age, sex, marital status and level of urbanization to the population of the north of the Netherlands at May 1^st^ 2012 was used. Marital status and level of urbanization were standardized for age and sex only.

### LifeLines versus POLS

The demographic characteristics of the standardized POLS sample resembled the total population (Dutch population register), which confirmed the expectation of a representative sample of POLS. Compared with POLS, individuals with a low educational attainment or without a paid job were underrepresented in LifeLines (standardized percentages were 4.8% and 28.8% in LifeLines versus 13.8% and 32.4% in POLS). LifeLines participants were more frequently overweight or obese (40.7% and 15.7% versus 35.8% and 12.2%; Table 2), but LifeLines participants had less frequently ever smoked (54.2% versus 66.0%). The prevalence of chronic diseases in both cohorts varied from 1.1% for stroke (in LifeLines) to 12.3% for chronic non-specific lung disease (in LifeLines). Except for osteoarthritis and chronic non-specific lung disease, differences in the prevalence of the various chronic diseases between LifeLines and POLS were 2.0% or smaller. Relative differences in the prevalence of diseases were larger and varied between 0% for cancer to 100% for stroke. Differences in all general health domains between the two populations were smaller than 5.5%.

**Table 2:** Socioeconomic characteristics, lifestyle, chronic diseases and general health in the LifeLines study population as compared with the population of the three northern provinces of the Netherlands.

	LifeLines		Permanent Survey of Living Conditions (sample)	
	Crude %	Standardized %	Crude %	Standardized %
N	151,481		6,093	
**Socioeconomic indicators**				
Elementary education	2,9	4,8	15,2	13,8
Secondary education	67,2	66,5	62,1	63,3
Tertiary education	30,0	28,8	22,6	22,8
No paid job	20,3	28,8	36,6	32,4
**Lifestyle** ^**a**^				
Overweight	39,0	40,7	37,1	35,8
Obese	15,6	15,7	12,7	12,2
Ever smoker	53,8	54,2	67,1	66,0
**Chronic diseases** ^**b**^				
Diabetes Mellitus	2,5	3,3	5,1	4,4
Stroke	0,8	1,1	2,6	2,2
Myocardial infarction	1,1	1,8	3,0	2,4
Cancer	4,6	5,7	6,9	5,7
Chronic non-specific lung disease	12,2	12,3	7,7	7,4
Osteoarthritis	7,3	8,9	13,2	11,5
Rheumatoid arthritis	2,2	2,8	5,3	4,8
Psoriasis	2,8	2,8	2,5	2,5
**General health**				
Less than good self-assessed health	9,7	9,6	10,3	9,4
Limited in moderate activities	14,7	16,8	24,1	22,1
Limited in stair climbing	21,4	23,3	22,4	20,4
Accomplished less due to physical problem	13,7	13,9	17,3	16,3
Limited in type of work/activities due to physical problem	13,7	14,1	18,1	16,8
Accomplished less due to emotional problem	10,8	10,7	10,6	10,6
Work activities less carefully due to emotional problem	9,4	9,2	10,2	10,1
Pain interfering with normal work	8,1	8,2	11,6	10,7

Direct standardization for age, sex, marital status and level of urbanization to the population of the north of the Netherlands at May 1^st^ 2012 was used.

^**a**^ Body- weight and height measured in LifeLines, self-reported in Permanent Survey of Living Conditions.

^**b**^ Occurrence ‘ever’ of diseases was assessed, except for Chronic non-specific lung disease, Osteoarthritis, Rheumatoid arthritis and Psoriasis in Permanent Survey of Living Conditions, for which the occurrence in last 12 months was assessed.

### Comparison of three recruitment strategies within LifeLines

Individuals in the index population were 25–49 years old, whereas a predominant part of the family members was younger than 25 years or older than 50 (Fig 1). Self-registrants were distributed more evenly across ages, which was more in line with the age distribution of the population of the three northern provinces of the Netherlands.

A higher percentage of females were observed among self-registrants (64.2%) than in the index population (57.9%) and family members (57.2%; Table 3). Also, relatively more self-registrants lived in an urban place (26.3%) compared to the index population (9.3%) and family members (15.6%). The percentage of western and non-western immigrants among family members (3.4% and 0.8%) was lower than in the index population (4.2% and 1.5%) or among self-registrants (4.8% and 1.6%). Compared with the index population or family members, the demographic characteristics of self-registrants resembled the population of the north of the Netherlands most closely, particularly for males (Fig 1).

**Table 3:** Demographic characteristics of the index population, family members and self-registrants of the LifeLines Cohort Study.

	Index population		Family members		Self-registrants	
	Crude %	Standardized %	Crude %	Standardized %	Crude %	Standardized %
N	81,840		48,366		21,275	
**Demographic indicators**						
Women	57.9		57.2		64.2	
18–24 years	0.1		14.7		8.2	
25–49 years	87.8		30.2		38.3	
50–74 years	12.1		51.9		51.7	
> = 75 years	0.1		3.3		1.9	
Married or registered partner	62.6	60.6	62.3	60.7	60.7	55.9
Urban municipality	10.5	9.3	15.1	15.6	24.4	26.3
Semi urban municipality	23.5	23.2	20.4	20.7	19.4	19.6
Rural municipality	66.0	67.6	64.5	63.8	56.2	54.1
Western migrant	4.4	4.2	3.3	3.4	4.9	4.8
Non-Western migrant	1.9	1.5	0.8	0.8	1.6	1.6

Direct standardization for age, sex, marital status and level of urbanization to the population of the north of the Netherlands at May 1^st^ 2012 was used. Marital status and level of urbanization were standardized for age and sex only.

Differences in the prevalence of socioeconomic indicators, lifestyle, chronic diseases and general health were generally small between the three groups (Table 4). Self-registrants had slightly less often a low educational attainment (3.7%) or no paid job (27.6%) than persons in the index population (4.3% and 28.5%) or self-registrants (4.9% and 29.2%). Relatively more participants in the index population had ever smoked (56.0%) compared to family members (54.5%) and self-registrants (53.9%). Overall, differences in measures of general health and the various chronic diseases between the three groups were small (<2.5%) and without a clear pattern.

**Table 4:** Socioeconomic characteristics, lifestyle, chronic diseases and general health in the index population, family members and self-registrants of the LifeLines Cohort Study.

	Index population		Family members		Self-registrants	
	Crude %	Standardized %	Crude %	Standardized %	Crude %	Standardized %
N	81,840		48,366		21,275	
**Socioeconomic indicators**						
Elementary education	1.8	4.3	4.7	4.9	3.0	3.7
Secondary education	66.5	65.8	69.6	67.3	64.3	63.9
Tertiary education	31.7	29.9	25.8	27.7	32.7	32.5
No paid job	11.7	28.5	31.9	29.2	26.5	27.6
**Lifestyle**						
Overweight	38.3	40.2	39.9	40.5	39.1	40.7
Obese	16.2	16.0	15.4	15.4	13.8	13.6
Ever smoker	52.4	56.0	54.5	52.9	56.8	53.9
**Chronic diseases**						
Diabetes Mellitus	1.7	3.8	3.9	3.4	2.8	2.7
Stroke	0.5	1.5	1.1	1.1	0.9	0.9
Myocardial infarction	0.5	1.8	1.9	2.0	1.2	1.3
Cancer	3.2	5.7	6.3	5.7	5.9	5.7
Chronic non-specific lung disease	12.1	12.5	12.4	12.3	12.6	12.5
Osteoarthritis	4.3	7.8	10.7	8.8	11.1	9.9
Rheumatoid arthritis	1.7	2.4	3.0	2.9	2.6	2.6
Psoriasis	2.9	2.9	2.8	2.8	2.8	2.8
**General health**						
Less than good self-assessed health	9.8	9.9	9.1	8.8	10.5	10.5
Limited in moderate activities	13.4	17.6	16.4	16.7	16.2	16.3
Limited in stair climbing	19.7	23.4	23.3	23.1	23.6	23.3
Accomplished less due to physical problem	14.0	15.5	13.1	13.6	14.2	14.3
Limited in type of work/activities due to physical problem	13.8	15.3	13.4	13.7	14.2	14.0
Accomplished less due to emotional problem	11.4	11.6	9.6	10.2	11.2	11.5
Work activities less carefully due to emotional problem	9.8	9.5	8.5	9.0	9.8	10.2
Pain interfering with normal work	8.1	8.8	7.8	8.0	8.5	8.2

Direct standardization for age, sex, marital status and level of urbanization to the population of the north of the Netherlands at May 1^st^ 2012 was used.

## Discussion

Middle aged individuals are overrepresented in the LifeLines Cohort Study, which is explained by the recruitment strategy adopted to recruit three generations of participants. Adjusted for demographic composition, LifeLines is broadly representative with respect to socioeconomic characteristics, lifestyle factors, the prevalence of chronic diseases and general health. Online self-registration yields a population that is more similar to the population in the north of the Netherlands as compared with recruitment via GPs or family members. Elderly persons appeared to participate less, which may be due to a higher rate of disabling diseases or less access to the internet for self-registration [14]. Less time to participate due to job related obligations and different beliefs regarding the importance of being healthy may explain the underrepresentation of men in LifeLines [15]. The smaller proportion of immigrants and persons with a low level of education or without a paid job in LifeLines may be explained by language related issues, a smaller trust in science and lower volunteerism [16–18]. Furthermore, the exclusion of persons who were unable to read Dutch by general practitioners during the recruitment period may also have contributed to the underrepresentation of immigrants. The smaller percentage of ever smokers may correlate with the lower participation rate among persons with a low educational attainment or may relate to fear for stigmatization when participating in health related research [17].

### Methodological considerations

We selected items that had good comparability in LifeLines and POLS, but some differences existed. In LifeLines, BMI was calculated using measured weight and height, whereas in POLS weight and height were self-assessed. Self-assessed BMI tends to be underreported, particularly by those who have a high BMI [19, 20]. In part, this may explain the larger proportion of overweight and obesity in LifeLines than in the POLS-survey. In LifeLines, the definition of ‘ever smoker’ was based on a question asking ‘Have you been a smoker for a complete year?’, whereas in POLS, the question was ‘Have you ever smoked some cigarettes?’ The more stringent definition in LifeLines may have led to a slight overestimation of the difference between LifeLines and POLS in the percentage of ‘ever smoker’. In LifeLines, individuals were asked to report whether they ever had chronic non-specific lung disease, osteoarthritis, rheumatoid arthritis and psoriasis, whereas in POLS it was asked to report the occurrence of the conditions in the past twelve months. Chronic non-specific lung disease is partly reversible, which may explain the higher percentage of individuals with the disease in LifeLines. The other conditions are largely irreversible.

Instead of systematically excluding all individuals with information missing on any of the variables, we chose to omit missing values for each item separately. To evaluate to what extent this choice affected our results, we performed an alternative analysis in which all individuals who had missing information on one or more items were excluded. This alternative analysis provided results similar to the original analysis, suggesting that this choice of handling missing values has not affected our substantive conclusions.

To obtain an impression of the extent to which our choice of comparing with the population in the north of the Netherlands as registered May 1^st^, 2012 has influenced our results, we performed a sensitivity analysis using the population as registered January 1^st^ 2006 as reference. Compared with the population in 2012, the population in 2006 was slightly younger, but other demographic indicators hardly differed. This suggests that the choice of reference date has not affected our results.

The POLS-survey does not include the institutionalized population, which has a worse health profile than the non-institutionalized population [21]. In the Netherlands, only a minor part of elderly people live in an institution, i.e.90% of those aged 80–85 still live at home [22]. Therefore, exclusion of the institutionalized population is not expected to have had a substantial impact on the representativeness of the survey. As the initial recruitment was done by GPs and the inclination to participate in a long term follow-up study in institutions is probably low, we do not expect many institutionalized persons to participate in LifeLines.

In our analysis, we deliberately chose to focus on absolute differences and not on statistical significance because the large study sample of LifeLines may produce low p-values even when absolute differences are small.

### Comparison with previous studies

In other cohort studies, women, and persons who are 80 years and older, not married, live in an urban place or are an immigrant were underrepresented [6,23,24]. Furthermore, persons with a low socioeconomic status or without paid work were less likely to participate [5,6,23–25]. Participants were less often a smoker, had a lower prevalence of chronic diseases, a better self-assessed health and less disability than non-participants [4–6,25]. These findings are in agreement with our results. In addition, our study shows that the inclusion strategy chosen may affect representativeness on demographic characteristics, but not so much on lifestyle, prevalence of chronic diseases and general health.

Previous studies have shown that a representative study sample can be achieved with various recruitment strategies including mass mailing, random digit dialing, internet recruitment, and recruitment via GPs [26,27]. For various reasons, in LifeLines, the initial recruitment was performed via GPs. In the Netherlands, GPs have a gatekeeping role, which means that patients can visit a hospital doctor only after referral by a GP. Therefore, GPs’ patient files cover basically the entire Dutch population. Furthermore, close involvement of GPs in LifeLines was essential because, when abnormalities were found, study participants were referred to their GPs and GPs were informed. Following the recruitment of the index population, family referral was used to include family members. Earlier studies showed that family referral not always results in a representative sample [28]. Our study suggests that a combined strategy of recruitment through GPs, family referral and self-registration leads to a broadly representative study sample.

### Implications

Unrepresentative study samples may challenge the generalizability of estimated relative risks [29]. This is particularly true when factors on which sample selection occurred are effect modifiers of the association under study [29]. We showed that, adjusted for differences in demographic composition, LifeLines is broadly representative on socioeconomic characteristics, lifestyle, diseases and general health. Although this is no strict guarantee against selection on other variables, it is a strong indication that LifeLines study population is a broadly representative sample of the population in the north of the Netherlands. This suggests that the risk of selection bias is low and that risk estimates are likely to represent associations in the population. Often, effect modifiers of a specific association under study are unknown or unmeasured. Also in this situation, when effect modification cannot be accounted for by subgroup analyses, a representative study sample is the best guarantee to obtain unbiased estimates [30–32]. We conclude that the LifeLines study population is broadly representative of the population in the north of the Netherlands and that the recruitment strategy has had no substantial effect on the representativeness.
